# Supplementary material for: Protective effect of plant compounds in pesticides toxicity
Source: J Environ Health Sci Eng. 2022 Sep 15;20(2):1035–45. doi: 10.1007/s40201-022-00823-0 (PMC9672277; doi:10.1007/s40201-022-00823-0)
Supplement: Supplementary file 1 — Supplementary Material 1 [file 40201_2022_823_MOESM1_ESM.docx]

**Pesticide-induced toxicity and the role of plant origin compounds in chemo-protection**

Agata Jabłońska – Trypuć^1^*, Józefa Wiater^2^

^1^Bialystok University of Technology, Faculty of Civil Engineering and Environmental Sciences, Division of Chemistry, Biology and Biotechnology, Białystok, Poland

Wiejska 45E Street, 15-351 Białystok, Poland

e-mail: a.jablonska@pb.edu.pl

^2^Bialystok University of Technology, Faculty of Civil Engineering and Environmental Sciences, Department of Agri-Food Engineering and Environmental Management, Białystok, Poland

Wiejska 45E Street, 15-351 Białystok, Poland

**Abstract**

**Introduction:** The relationship between pesticide exposure and the occurrence of many chronic diseases, including cancer, is confirmed by literature data.

**Methods:** In this review, through the analysis of more than 70 papers, we explore an increase in oxidative stress level caused by exposure to environmental pollutants and the protective effects of plant-origin antioxidants.

**Results and Discussion:** One of the molecular mechanisms, by which pesticides affect living organisms is the induction of oxidative stress. However, recently many plant-based dietary ingredients with antioxidant properties have been considered as a chemopreventive substances due to their ability to remove free radicals. Such a food component must meet several conditions: eliminate free radicals, be easily absorbed and function at an appropriate physiological level. Its main function is to maintain the redox balance and minimize the cellular damage caused by ROS. Therefore, it should be active in aqueous solutions and membrane domains. These properties are characteristic for phenolic compounds and selected plant hormones. Phenolic compounds have proven antioxidant properties, while increasing number of compounds from the group of plant hormones with a very diverse chemical structure turn out to act as antioxidants, being potential food ingredients that can eliminate negative effects of pesticides.

**Key words**: pesticides, oxidative stress, toxicity, polyphenols, cytokinins

**1. Introduction**

Pesticides consist a very large and diverse group of chemical compounds and their mixtures. They are important from the environmental protection point of view, because they can disturb the natural balance of ecosystems. Problems with the use of pesticides are often associated with agriculture, forestry, and their accumulation as a result of spraying weeds growing along roads, railway lines, in gardens, parks and other urban areas [1]. Given their specific properties, i.e., the ability to bioaccumulation and the high persistence in ecosystems, it seems obvious that their residues are found in living organisms [2] (Fig.1). In order to estimate their effects in the environment, it is important to analyze their bioconcentration resulting from the exposure to these compounds in the diet [3]. The term "bioconcentration" commonly used to describe the penetration and metabolism of pesticides in living organisms contains three important aspects: physicochemical properties of individual chemical compounds that are active substances of pesticides, physiological dispositions of the penetrated organism and environmental conditions surrounding the body. Pesticides can enter living organisms through various pathways, including: absorption through the skin, inhalation and through the digestive tract [4]. There are many reports confirming the relationship between exposure to pesticides and the occurrence of many chronic diseases such as various types of cancer, diabetes, neurodegenerative diseases, birth defects, endocrine disorders, respiratory problems, asthma, cardiovascular diseases, nephropathy, autoimmune diseases, chronic fatigue syndrome and aging [5,6,7]. One of the main molecular mechanisms, by which pesticides affect living organisms influencing their health, is an increase in oxidative stress level.

Due to the inevitable exposure of humans to pesticides, which are common environmental toxins, there is a need for effective methods to reduce, and preferably eliminate, their harmful effects. Complementary and alternative solutions in the form of dietary antioxidants or functional foods can provide a safe way to prevent pesticide toxicity. In recent years, many plant derived dietary compounds, especially with antioxidative activity attract attention as chemopreventive substances, because of their ability to scavenge free radicals generated by environmental contaminants. Popular group of plant-derived compounds are flavonoids, which comprise a large, heterogenous group with benzopyran in their structure. They are present in vegetables, fruits and herbs as a secondary plant metabolite. They have free radical scavenging abilities and exhibit positive health effect, especially in cancer treatment and prevention [8]. Many compounds from the large group of plant hormones are tested for their antioxidative properties in human organism likewise. An example of such group of compounds are cytokinins, and well-known compound from this group considering its influence on oxidative stress parameters is kinetin. In human cells it acts as an antioxidant with chemo-protective properties [9].

The mechanisms, by which pesticides may cause damage both on the cellular and tissue level usually involve oxidative stress reactions, especially generation of free radicals, lipid peroxides, oxidized proteins and oxidized sugars [10,11]. Cellular enzymes, structural proteins, membranes components, sugars and nucleic acids are exposed to possible damage because of ROS (Reactive Oxygen Species) high reactivity [12,13]. The stimulatory influence of pesticides on lipid peroxidation and protein, DNA and sugars oxidation processes has been well documented, particularly in vivo [14,15]. However, mechanisms, by which pesticides act on the cellular and molecular level are still not fully defined. Therefore, it is very important to study this phenomenon on a variety of levels and in many possible combinations, because the activity of pesticides in cells varies depending on their chemical structure, dose and time of exposure.

The objective of this review is to make a state-of-the-art overview of food contaminants and beneficial food components mechanisms of activity and their mutual interactions. For this purpose, a literature review was carried out focusing on new reports in the field of oxidative stress definition and parameters, pesticides as oxidative stress inductors and plant-derived compounds as antioxidants. More than 70 papers published since 1937 were analyzed with focusing mainly on the last 10 years articles considering mainly oxidative stress, the influence of pesticides on oxidative stress parameters on the cellular level and possible chemo-preventive activity of polyphenols and selected plant hormones. This review will discuss the potential benefits of polyphenolic compounds and plant hormone compounds in alleviating the side effects and toxicity associated with common environmental toxins such as pesticides, primarily in in vitro studies.

The importance of this review is that it mainly aims to determine whether polyphenol compounds and cytokinins can modulate the toxicity of environmental pollutants, thereby affecting health and the possibility of diseases generated by oxidative stress. The phenomenon of oxidative stress and its causes will be explained. Evidence will be provided that pesticide pollution increases oxidative stress level and plant-derived antioxidants may play a role in neutralizing or buffering the effects of oxidizing pollutants.

This review summarizes also the most common and health-relevant sources of oxidative stress, with an emphasis on pesticides and the potential to counter them with substances of plant origin. Due to extensive scientific data, not all studies could be included in this review. The reader is referred to the analysis of the references presented in the paper (and the references contained therein) in order to obtain details on the pro-oxidative action of pesticides and the antioxidant action of selected compounds of plant origin.

Fig.1. The links between pesticides origin from various sources (industrial and agricultural), reactive oxygen species and health outcomes.

**2. Selected pesticides influence on oxidative stress**

**2.1. Pesticides characteristic**

Pesticides constitute a group of compounds widely used all over the world in agriculture and many other industries. In European Union, only for plant protection, more than 140 000 tons of pesticides are being used every year. Chemical compound used as a pesticide should be characterized by high toxicity towards pests and simultaneously possibly low toxicity towards other organisms, especially human and aquatic organisms. They also should be accordingly durable, susceptible to biodegradation to such an extent that they do not accumulate in the environment [16]. There are many categories according to which pesticides can be divided, e.g., depending on the organism to be combated, they can be divided into zoocides (combating animal pests), bacteriocides (against bacteria), herbicides (against weeds) and fungicides (against fungi). The division in terms of chemical structure can be also applied. Main groups are nonorganic pesticides (arsenic and fluoride insecticides) and organic pesticides (chloroorganic, phosphoroorganic, carbaminianes, phenoxyacetic acid and triazyne derivatives). Increasingly common use of pesticides even in food storage is a main reason why people are constantly exposed to their residues in food products and potable water. Only a part of pesticides used in EU is under the monitoring system and almost 50% of fruits, vegetables and crops is contaminated with pesticides and their residues [17]. According to WHO pesticide residues in food are defined as a sum of chemical compounds present in food product as a result of pesticide usage (the parent substance and its degradation products). In addition to pesticides, smoke, mycotoxins, endocrine disrupting chemicals (such as polychlorinated biphenyl) and heavy metals (such as arsenic) have been listed as the most common human toxins. Toxins are poisons produced in living organisms of plants, animals and bacteria. Toxic substances (toxicants) are synthetic, toxic, man-made chemicals. These two groups of compounds also differ in the processes of production, distribution, heterogeneity of composition and increasing ubiquity at homes, in human and animal bodies and in the environment. They can cause biochemical damage in various ways. While there are many types of toxins, they tend to interfere with the normal activity of cells, making cells act differently than normal [18]. Carcinogens, such as pesticides, cause the multiplication of various cells to accelerate and cause cancer. Instead of disrupting certain processes in the body, many poisons, including pesticides, cause them to act differently, often with harmful effects. People deal with toxins with antidotes, either by building up immunity or by avoiding them. However, toxic substances such as pesticides exist on a massive scale and are associated with everyday economic, industrial and regulatory systems. This means that efforts to mitigate or eliminate the effects of pesticides must take into account these systems and the power dynamics that maintain them. One of the main factors connected with the risk that pesticides constitute to human health is their dose of exposure [19]. Three categories of pesticide exposure can be distinguished: intentional (accidental/suicidal), occupational and non-occupational exposure [20]. An intentional exposure is usually considered as a possibility to attempt suicide in developing countries, because of an easy access to such chemicals especially in rural areas in China and South East Asian countries [21]. The second kind of pesticide exposure – occupational, is mainly considered as connected with pesticides transport and usage in crop protection, food storage and food sale. People such as dealers, farmers, applicators and sellers of fruit and vegetables are particularly exposed to pesticides, especially while mixing, loading, spraying and through a direct contact with fruits, vegetables and crops. In this case pesticides enter human organism through the respiratory tract and the skin [22]. The third kind of pesticide exposure is non-occupational exposure, which is the most common way of the exposition. It occurs through the gastrointestinal tract with the consumption of fruits, vegetables and crops. It is rather difficult to exactly demonstrate the detailed cause and effect relationship associated with pesticide exposition and their possible hazardous effect. It should be also mentioned that the literature data based on the mammalian experimental studies, which connect directly pesticides toxicity and the mechanisms of their action, especially oxidative stress, are very scarce.

**2.2. Oxidative stress definition, sources and consequences**

Oxidative stress is considered as a kind of phenomenon, which is related to the activity of free radicals (FR) and reactive metabolites (RM) in the human organism. Free radicals originate from different sources and occur in human cells as a product of normal cellular metabolism. They have double functions: as beneficial and toxic compounds (Fig 2) [23,24] Low concentration of free radicals exerts beneficial effect on human metabolism. It results from their involvement in a variety of physiological functions such as immune system activity, cellular signaling pathways, mitogenic response and redox regulation [25]. Complete suppression of free radicals’ generation is not preferred under physiological conditions, because their certain amount is required for proper cell function [26]. However, too high concentration of FR, both reactive oxygen species (ROS) and reactive nitrogen species (RNS) is a major cause of oxidative and nitrosative stress. It subsequently may cause a potential damage to important biomolecules such as proteins, lipids, sugars and nucleic acids [27]. In this sense oxidative stress is a kind of an imbalance between FR generation and antioxidants production, which leads to the damage on the cellular and tissue level [28]. Prooxidants are endobiotic or xenobiotic compounds that induce oxidative stress by the generation of ROS and/or an inhibition of an antioxidant system (Fig 3). However, it should be also mentioned that oxidation process never occurs alone, it is always supported by reduction process. Therefore, a definitely more precise term should be used for the description of this phenomenon – “redox stress” [23]. Living organisms have built protective systems against the excess of free radicals resulting in lowering of their toxic activity. The first level of the antioxidant defense is the prevention against FR formation. It includes the presence of the inhibitors of enzymes catalyzing generation of FR. An example of such an enzyme can be xanthine oxidase. If free radicals have already been formed it means that primary protection is insufficient. In that situation FR scavengers are involved and they convert FR into nontoxic compounds. These are antioxidants, which prevent the oxidation of biologically important macromolecules (Fig 4). The third level of the defense against FR damage is repair system, which plays an important role in the identification, repair and disposal of damaged molecules. An example is the activity of proteinases, which remove damaged proteins; lipases, which remove oxidatively damaged lipids and DNA repair system. Actually, a growing body of evidence shows that oxidative stress is one of the major causes of many acute and chronic human diseases, such as: diabetes, neurodegenerative diseases (Alzheimer’s Disease, Parkinson’s Disease, Multiple Sclerosis), cardiovascular diseases (atherosclerosis, hypertension), cancer, cataracts, asthma, rheumatoid arthritis, inflammation, burns, intestinal tract diseases, progerias and others [24, 29-32]. Above mentioned diseases appear as a result of prolonged or intensified oxidative stress, which leads to permanent changes in the structure of biologically important macromolecules, such as DNA, proteins, lipids and sugars. In turn, changes in the structure of molecules lead to changes in their functioning, which is manifested in the impairment of cellular metabolism. DNA stability is a prerequisite for the proper functioning of cells because its damage may lead to disturbance of cellular metabolism and to the development of many diseases, including cancer. More susceptible for ROS attack is rather mitochondrial DNA than nuclear DNA, because of its location adjacent to the cellular place where ROS are generated. Free radicals, especially OH•, very easily react with all of the constituents of DNA: purine and pyrimidine bases, deoxyribose sugar causing a number of alternations [33]. Among others these are damages to single nitrogen bases, DNA strand breaks or adduct formation.

Fig 2. Sources of free radicals in human environment

Fig 3. Classification of prooxidants in human environment

Fig 4. Classification of antioxidants occurring in living organisms and available from the dietary sources.

**2.3. Examples of pesticides as oxidative stress inducers**

Laboratory studies are focused on clarifying mechanisms of pesticides action on the cellular level. A growing body of evidence indicates that pesticides’ toxicity may be connected with an induction of oxidative stress. Long-term and severe oxidative stress is very harmful for cells because it causes metabolic disorders. The overproduction of ROS may also be caused by the influence of the pesticides on the activity of the endoplasmatic reticulum and mitochondrial electron transport chains. The other mechanism, which has been proposed as an explanation for the toxic activity of pesticides, is an increase in ROS generation by pesticides entering redox cycles, e.g., autooxidation. According to the literature data pesticides may inhibit antioxidant enzymes activity and the biosynthesis of selected antioxidants such as glutathione [10,11,34,35]. From the group of herbicides, the most widely known and used is paraquat (PQ), which causes severe damage in diverse organs including lungs, especially in case of ingestion accidentally or intentionally (Fig.5). In paraquat toxicity ROS generation is involved. Kanno S. et al. examined the effect of paraquat on two lung epithelial cell lines: A549 and BEAS-2B. They found out that PQ toxicity at the cellular level is connected with mitochondrial dysfunction due to the cellular accumulation of PQ more than ROS generation [36]. Additionally, Jaroonwitchawan T et al reported that paraquat is a neurotoxic agent, which causes neuronal cell death through the induction of oxidative stress level. However, they also stated that curcumin is a compound with potentially therapeutic properties in case of neurodegenerative diseases associated with ROS overproduction induced by pesticides [37]. According to Dou T. et al PQ is a strong inducer of oxidative stress in immortalized human embryonic neural progenitor cells. It caused significant decrease in the activity of SOD and CAT but increase in MDA and LDH level. Authors also revealed that Nrf2/ARE pathway is involved in PQ-induced oxidative stress [38]. Wang S et al. found out that atrazine in fish induces apoptosis through the activation of caspase 3 and through the changes in the expression of mitochondrial pathways factors (Bcl-2, Bax, caspase 9) and death receptor pathways (TNF-α, TNFR, Fas, FasL, and Caspase 8) (Fig.5). Tested herbicide also inhibited antioxidative enzymes activity, increased ROS level, lowered GSH content and enhanced MDA accumulation. It means that the oxidative stress and mitochondrial damage caused by atrazine metabolism may play a crucial role in the apoptosis of carp neutrophils [39]. Ansari SM et al examined the effect of pendimethalin (PM) - dinitroaniline herbicide extensively applied against the annual grasses and broad-leaved weeds, on human primary cells (Fig.5). They found out that PM induces genotoxic and apoptotic changes and influences antioxidant enzymes activity in human lymphocytes and rat bone-marrow cells [40]. According to Degl'Innocenti D et al oxadiazon, a pre-emergence or early post-emergence herbicide, could be considered as a potential cause of the onset of neurodegenerative diseases (Fig.5). Their findings reveal that oxadiazon influences activity of the mitochondrial aldehyde dehydrogenase 2 (ALDH2) and of the acylphosphatase (ACYP). ALDH2 activity is connected with the protection of neurons against oxidative stress caused by toxic aldehydes. ACYP activity on the other hand is connected with cell differentiation, apoptosis and cancer. Both the enzymes are important factors considered in neurodegenerative conditions such as Alzheimer’s and Parkinson's disease [41]. It was also confirmed that oxadiazon may induce liver cancer and causes adverse effects on reproductive and on endocrine functions [42,43].

Fig.5. Selected pesticides exhibiting pro-oxidative properties in human and animals’ organisms consisting threat to human/animals’ health

**3. Plant origin substances as antioxidants**

Nowadays we observe an urgent need to deepen and develop our knowledge regarding food contaminants and food ingredients, both in vitro and in vivo. As it was already mentioned, pesticides belong to the group of highly undesirable food contaminants, which cause serious health problems. On the other hand, food, especially natural and plant derived food, contains a lot of ingredients with very beneficial for human health properties, such as polyphenols with proved antioxidative capacities or plant hormones – a very diverse in terms of chemical structure, group of compounds. Recognizing the fact that pesticides induce their toxic effects via oxidative stress pathways and stimulation of the inflammatory response, mainly antioxidants and anti-inflamatories have been studied as potential therapeutic strategies. Antioxidants are compounds, which delay or inhibit oxidative damage, because they are stable enough to neutralize free radicals by donating electrons. In human organism a highly complex antioxidant system is developed, and it includes both enzymatic and non-enzymatic part. Because of its synergistic activity it can protect cells, tissues and organs against FR damage. Endogenous antioxidants create a widely distributed within the cytoplasm and various organelles network [44]. Natural, food-derived compounds have recently received a great attention, because of their ability to neutralize free radicals and/or to enhance endogenous activity of antioxidative system. Food-derived candidate for an ideal antioxidant should meet several conditions: it should easily eliminate free radicals and be readily absorbed, it should have the ability to chelating redox metals and acts on at physiologically appropriate level. Its main function is maintaining a delicate redox balance and minimizing cellular damage caused by ROS, therefore it should also be active in aqueous solutions and membrane domains. Dietary antioxidants are required for maintain normal cellular functions when endogenous antioxidative system is insufficient, e.g., under conditions, which support oxidative stress.

**3.1. Amelioration of pesticide induced oxidative stress by polyphenols**

Polyphenols belong to the group of natural compounds of plant origin that are characterized by a variety of biological activities. They may react with ROS and therefore terminate chain reactions before cell metabolism is seriously affected [45]. Polyphenols are present in many different dietary compounds such as fruits (e.g., apples, grapes, pears, cherries and berries), vegetables, (beverages (e.g., red wine, tea, coffee), chocolate, cereals, dry legumes, herbs, spices, flowers [46,47]. Polyphenols content in food is rather higher than any other food ingredient with antioxidative properties. They are plant metabolites with specific properties that allow them to act as protection against UV radiation and damage caused by pathogens [48]. Their content in plants depends on many environmental factors, including the type of soil, exposure to light, intensity and frequency of rainfall and methods of plant breeding. The group of phenolic compounds includes about 8,000 compounds, including: flavanols, flavones, isoflavones, tannins, resveratrol, anthocyanidins, curcumin, lignans and phenolic acids. Their bioavailability depends on many different parameters, such as digestion, absorption, metabolism [49].

The antioxidant properties of phenolic compounds of natural origin are the subject of many studies and reports, but the molecular mechanism of their action is not fully understood. It is a very complex group of compounds that differ in their chemical structure, which also affects their activity at the cellular level. The main mechanisms, by which, plant phenolic compounds act at the molecular level are primarily their action as so-called " free radicals’ scavengers", their ability to chelate metals and to inhibit various types of oxidases (lipoxygenases, cyclooxygenases, etc.) and the stimulation of the activity of antioxidant enzymes, such as catalase, superoxide dismutase, etc. [50,51].

The antioxidant activity of phenolic compounds has been demonstrated in both in vitro and in vivo tests. Bioactive components of green tea extracts, including phenolic compounds such as EGCG (epigallocatechin gallate) reduce the level of free radicals in healthy cells in vitro at the concentration range from 10 µM to 20 µM (Fig.6). However, in higher concentration (100 µM), they generate ROS in neoplastic cells [52]. Literature data show that EGCG has the ability to inhibit pesticide-induced apoptosis [53,54]. Kamalden et al. found that green tea phenolic compounds reduce the level of lipid peroxidation and rotenone toxicity in RGC-5 cells [55]. In addition to in vitro studies, animal studies have also confirmed the beneficial effects of green tea extracts. These rich in polyphenol mixtures reduce the toxic effects of pesticides, including oxidative stress in the liver, lungs and nervous system [56,57]. It was observed that under the influence of the tested pesticides changes in the parameters of oxidative stress occurred. It was: an increase in the activity of enzymes such as SOD, catalase, decrease in GSH content, increase in the level of lipid peroxidation, which indicated an increased level of oxidative stress. Supplementation with green tea extracts alleviated degenerative processes in the liver, reduced hepatocyte necrosis and normalized the levels of antioxidant enzyme activity in rats. Kim et al. demonstrated that green tea extracts rich in polyphenols significantly reduced pulmonary fibrosis caused by pesticide-induced oxidative stress [57]. Components of green tea extracts supposedly acted to suppress oxidative stress and endothelin-1 expression [57]. On the other hand, another compound from the group of phenolic compounds - resveratrol - acts in the opposite way to EGCG. At lower concentrations, it induces an increase in the number of free radicals in cancer cells, while at higher concentrations it inhibits their generation. It similarly influences the activity of antioxidant enzymes, such as superoxide dismutase or catalase. Resveratrol has anti-inflammatory and anti-cancer effects by modulating oxidative stress and influencing glucose levels in cancer cells. Inhibition of ROS synthesis in neoplastic cells may contribute to their death. They are usually characterized by an increased level of oxidative stress in relation to healthy cells. Therefore, resveratrol inhibits the growth of neoplasms by inhibiting intracellular ROS and suppressing glycolytic metabolism of neoplastic cells [58].

Curcumin is a polyphenolic compound, extracted from the very popular spice obtained from turmeric (Curcuma longa), which also has antioxidant and anti-inflammatory properties (Fig.6). Its anti-cancer effect is also associated with the mechanisms of oxidative stress. It induces phase II antioxidant enzymes by activating the NRF2 signaling pathway, activating the p53 suppressor protein and modulating inflammatory mediators such as TGF-β and COX2 [59]. Probably due to excellent antioxidant properties and strong action of eliminating free radicals at the cellular level, phenolic compounds are tested as potential food ingredients reducing the toxic effects of pesticides [60, 61, 62].

Fig.6. Selected compounds from the group of polyphenols exhibiting antioxidative properties.

**3.2. Amelioration of pesticide – induced oxidative stress by selected plant hormones**

One of the most important group of plant hormones are cytokinins, which were identified while searching for factors responsible for cell division in plant in vitro cultures. It is a group of hormones that influence seed germination, chloroplast differentiation, flower development, apical dominance, leaf aging and interactions with pathogens. They also accelerate regenerative processes and extend the cell cycle [63]. In terms of chemical structure, cytokinins are a very complex group. The vast majority consists derivatives of adenine, in which an aliphatic or aromatic substituent is attached to the amino group of the C-6 carbon. An example of a cytokinin with an aliphatic substituent is zeatin, and with an aromatic substituent - benzyladenine. However, the best known cytokinin so far is kinetin, which for long time has been considered as a synthetic compound (Fig.7). It has been found in plant cells and even in human urine and in DNA extracted from human cells. Kinetin biosynthesis is called "free radical sink", which means that it is considered as a route of elimination of harmful products of metabolism. In particular these are the products of free radical reactions; therefore, kinetin constitutes a defense mechanism generated in response to oxidative stress [64]. Due to its antioxidant properties, kinetin can be considered as a compound that minimizes the negative impact of pesticides on the human body. Its antioxidant activity has been confirmed both in in vitro and in vivo tests. Kinetin is protective against cellular macromolecules such as DNA through at least two mechanisms. It can both prevent the formation of the hydroxyl radical and absorb free radicals before they can react with the DNA. The kinetin binds the iron ion required in the Fenton reaction, preventing the iron from binding to the nucleic acid. Elimination of free radicals with the participation of kinetin can also take place in two ways. Free radicals can split the hydrogen atom from the carbon atom of the amine bond of kinetin or undergo dismutation due to the fact that the kinetin in a complex with Cu (II) acts as a SOD mimetic [65]. Another cytokinin also initially considered as a synthetic compound is benzyladenine (Fig.7). However, it has been identified in many plant species, where it exhibits a strong anabolic effect [66]. Benzyladenine and its derivatives regulate many important cellular processes related mainly to cell division, melanogenesis and inflammatory reactions, where they influence the activity of phosphodiesterases [67,68,69]. Among the mechanisms proposed to explain the biological activity of N6-benzyladenine is its ability to bind to protein and influence the activity of antioxidant enzymes through competitive or allosteric interactions. Among them regulation of signal transduction; modulation of redox-sensitive transcription factors, including Nrf2, NF-κB, and AP-1; glutathione biosynthesis and gene expression can be mentioned. N6-benzyladenine binds a specific receptor, activates the kinase cascade, and consequently causes specific gene activation [70]. In vitro studies on the effect of kinetin and N6-benzyladenine on human fibroblasts showed the stimulating effect of both tested compounds on the activity of antioxidant enzymes, as well as on the content of reduced glutathione and thiol groups. Cytokinins reduced the peroxidation of membrane phospholipids and showed protective properties against malonyldialdehyde production. Both N6-benzyladenine and kinetin are characterized by complex mechanisms of action in fibroblast cells in vitro [9]. Both compounds have antioxidant properties and are potentially potent agents for use in the prevention and treatment of many diseases related to pesticide-induced oxidative stress.

A particularly interesting compound from the cytokinin group, which so far has not received much attention in research, is traumatic acid and its aldehyde derivative - traumatin. Traumatic acid (TA, trans-2-dodecenedioic acid) belongs to the derivatives of unsaturated fatty acids (Fig.7). It was first isolated from unripe bean pods (*Phaseolus vulgaris*) in 1937–1938 by Bonnier and English. So far, its presence has been detected in mesophyll and meristematic tissues of many plant species. Traumatic acid, as well as its aldehyde derivative - traumatin are called wound healing hormones, because they are present in large amount around the damaged area in plants and stimulate cell division. Both compounds are found in the highest amount in young, intensively growing organs, such as leaves, fruits and seeds. It turned out that traumatin, the aldehyde form, is a more biologically active compound than traumatic acid in plants. TA as a compound of plant origin may be an alternative to fatty acids of animal origin [71,72]. So far, there is very little literature data on the influence of traumatic acid on the parameters of oxidative stress, although the available information indicate that it is related to a high antioxidant and anticancer potential. The available studies have shown that it stimulates the activity of antioxidant enzymes and enhances other non-enzymatic parameters of oxidative stress in healthy human cells in vitro, while in tumor cell lines it acts the opposite, showing high toxicity. The stimulating effect of TA on the basic parameters of oxidative stress, such as the activity of antioxidant enzymes, the concentration of reduced glutathione, the content of thiol groups and the level of the lipid peroxidation process under physiological conditions, is presented. TA caused a decrease in peroxidation of membrane phospholipids and showed protective properties against ROS production [73]. On the other hand, in cancer cells it’s toxicity manifests by inducing oxidative stress and triggering apoptosis. Because of the proven relationship between dietary fat consumption and breast cancer incidence, the MCF-7 breast cancer cell line has been an experimental model. TA caused a decrease in cell proliferation and viability, a decrease in the content of reduced glutathione, which is the main low-molecular antioxidant in cells, and a decrease in the level of thiol groups. At the same time, it increased caspase 7 activity, peroxidation of membrane lipids and ROS content. The pro-apoptotic effect of TA can be attributed to the observed decrease in GSH levels and an increase in the level of oxidative stress. It has been suggested that a high level of GSH in breast cancer tissue is necessary to ensure cell proliferation and resistance to apoptosis in cancer cells [74]. TA induced a significant decrease in cell proliferation and an increase in caspase levels, accompanied by a decrease in GSH content and an increase in ROS content [75]. The extremely favorable properties of traumatic acid, observed in vitro, encourage the study of its possible interactions with pesticides. Such models of in vitro tests indicate the possibility of using TA as a factor eliminating the carcinogenic effects of selected pesticides. To analyze the potential effects of selected herbicides (MCPA, mesotrione, bifenox and dichlobenil) on cancer cells, four pesticide cytotoxicity studies were performed and a combination of TA with herbicides was used on three different breast cancer cell lines (MCF-7, ZR-75-1 and MDA- MB-231) and one healthy cell line MCF-12A. It has been found that in vitro exposure to various herbicides can increase the proliferation of breast cancer cells, leading to possible tumor growth and development, and TA can attenuate pesticide-induced cell stimulation, and it is not toxic to normal cells [76]. However, only clarification of the mechanisms of interaction of traumatic acid and pesticides will allow us to answer the question whether oxidative stress and changes in its parameters are involved in the interaction of compounds at the cellular level. The research conducted on the ZR-75-1 line on the activity of TA in combination with the herbicide - mesotrione, allow to conclude that TA contributes to the inhibition of growth and development of mesotrione-stimulated ZR-75-1 cells by stimulating oxidative stress and apoptosis. This may mean that TA is a compound with pro-oxidative and pro-apoptotic effects in neoplastic cells whose development and proliferation are stimulated by the presence of mesotrione [77]. The presented results may be helpful in answering the question of whether herbicides and their residues present in food may pose a potential threat to people with diagnosed cancer and whether compounds with proven pro-oxidative effects on cancer cells may have potential cytoprotective functions.

Fig.7. Selected compounds from the group of cytokinins, characterized by antioxidative properties.

**4. Conclusions**

The increasing presence of pesticides and their residues in food seems to be an inevitable problem that humanity will face for many years to come. On the other hand, evidence is emerging that shows a promising protective and detoxifying effect of plant-derived compounds such as polyphenols and cytokinins on environmental toxins. However, in many cases, the lack of clinical evidence for the efficacy of selected compounds adds to the need for such future research. For example, the development of predictive biomarkers of polyphenol and cytokinin intake in the human population will provide a better understanding of the interactions between these compounds and endogenous and exogenous factors that affect their bioavailability, and help determine safe dosages for their consumption. Interactions of plant-based dietary ingredients with pesticides and compounds with similar toxin modulating effects may also warrant further research.

Most people maintain a stable level of oxidative stress, and no matter how much additional antioxidant people ingest in their diet, oxidative stress does not decrease further. Antioxidants appear to be effective in reducing oxidative stress when its initial level is above normal or above a stable regulated level. Thus, additional dietary antioxidants can only benefit the body if it was necessary to correct high levels of oxidative stress that could not be controlled by endogenous antioxidants.

Finally, it should be emphasized that more research needs to be done to strengthen the evidence of the use of dietary antioxidant components as modulators of adverse effects caused by increased exposure to pesticides.

**Declarations**

**Funding**: This work was financially supported by National Science Centre (NCN), Poland, under the research project number 2021/05/X/NZ7/00433

**Conflicts of interest/Competing interests**: The author declare no conflict of interest

**Availability of data and material**: Not applicable

**Code availability**: Not applicable

**Ethics approval**: Not applicable

**Authors ‘contributions:** equal **Consent for publication:** we, authors give the consent for publication

**5. References**

1. Lushchak VI, Matviishyn TM, Husak VV, Storey JM, Storey KB. Pesticide toxicity: a mechanistic approach. EXCLI J. 2018;17:1101-1136.

2. Bolognesi C, Merlo FD. Pesticides: human health effects. In: Nriagu JO, editor. Encyclopedia of environmental health. Burlington: Elsevier; 2011. pp. 438–453.

3. Katagi T. Bioconcentration, bioaccumulation, and metabolism of pesticides in aquatic organisms. Rev Environ Contam Toxicol. 2010;204:1–132.

4. Jabłońska-Trypuć A, Wołejko E, Wydro U, Butarewicz A. The impact of pesticides on oxidative stress level in human organism and their activity as an endocrine disruptor. J Environ Sci Health B. 2017;52(7):483-494.

5. Abdollahi M, Ranjbar A, Shadnia S, Nikfar S, Rezaie A. Pesticides and oxidative stress: a review. Med Sci Monit. 2004;10 (6): 141-147.

6. De Souza A, Medeiros Ados R, De Souza AC, Wink M, Siqueira IR, Ferreira MB, Fernandes L, Loayza Hidalgo MP, Torres I.L. Evaluation of the impact of exposure to pesticides on the health of the rural population: Vale do Taquari, State of Rio Grande do Sul (Brazil). Cien Saude Colet. 2011;16 (8):3519-3528.

7. Mostafalou S, Abdollahi M. Concerns of environmental persistence of pesticides and human chronic diseases. Clin Exp Pharmacol. 2012;2 (3):1000e1108.

8. Rice-Evans CA, Miller NJ, Bolwell PG, Bramley PM, Pridham JB. The relative antioxidant activities of plant-derived polyphenolic flavonoids. Free Radic Res. 1995;22(4):375-83.

9. Jabłońska-Trypuć A, Matejczyk M, Czerpak R. N6-benzyladenine and kinetin influence antioxidative stress parameters in human skin fibroblasts. Mol Cell Biochem. 2016;413(1-2):97-107.

10. Wang X, Martínez MA, Dai M, Chen D, Ares I, Romero A, et al. Permethrin-induced oxidative stress and toxicity and metabolism: A review. Environ Res. 2016;149:86–104.

11. Lushchak V, Semchyshyn H, Lushchak O, Mandryk S. Diethyldithiocarbamate inhibits in vivo Cu,Zn-superoxide dismutase and perturbs free radical processes in the yeast Saccharomyces cerevisiae cells. Biochem Biophys Res Commun. 2005;338:1739–1744.

12. Jaganjac M, Cacev T, Cipak A, Kapitanović S, Gall Troselj K, Zarković N. Even stressed cells are individuals: second messengers of free radicals in pathophysiology of cancer. Croat Med J. 2012;53(4):304-9.

13. Zarkovic N. Antioxidants and Second Messengers of Free Radicals. Antioxidants (Basel). 2018;7(11): E158.

14. Semren TŽ, Žunec S, Pizent A. Oxidative stress in triazine pesticide toxicity: a review of the main biomarker findings. Arh Hig Rada Toksikol. 2018;69(2):109-125.

15. Slaninova A, Smutna M, Modra H, Svobodova Z. A review: oxidative stress in fish induced by pesticides. Neuro Endocrinol Lett. 2009;30 Suppl 1:2-12.

16. Bjørling-Poulsen M, Andersen HR, Grandjean P. Potential developmental neurotoxicity of pesticides used in Europe. Environ Health. 2008;7:50.

17. Monitoring of Pesticide Residues in Products of Plant Origin in the European Union, Norway, Iceland and Liechtenstein, 2005. Commision staff working document http://ec.europa.eu/food/fvo/specialreports/pesticide_residues/report_2005_en.pdf(19.05.201 )

18. Duke SO, Cantrell CL, Meepagala KM, Wedge DE, Tabanca N, Schrader KK. Natural toxins for use in pest management. Toxins (Basel). 2010;2(8):1943-1962.

19. Sabarwal A, Kumar K, Singh RP. Hazardous effects of chemical pesticides on human health-Cancer and other associated disorders. Environ Toxicol Pharmacol. 2018;63:103-114

20. Davies JE, Freed VH, Enos HF, Barquet A, Morgade C, Danauskas JX. Minimizing occupational exposure to pesticides: epidemiological overview. Residue Rev. 1980;75: 7–20.

21. Bertolote JM, Fleischmann A, Eddleston M, Gunnell, D. Deaths from pesticidepoisoning: a global response. Br J Psychiatry J Ment Sci. 2006;189: 201–203.

22. Shadnia S, Azizi E, Hosseini R, Khoei S, Fouladdel S, Pajoumand A, Jalali N, Abdollahi M. Evaluation of oxidative stress and genotoxicity in organophosphorus insecticide formulators. Hum Exp Toxicol. 2005;24(9):439-45.

23. Ďuračková Z. Some current insights into oxidative stress. Physiol Res. 2010;59(4):459-469.

24. Phaniendra A, Jestadi DB, Periyasamy L. Free radicals: properties, sources, targets, and their implication in various diseases. Indian J Clin Biochem. 2015 Jan;30(1):11-26.

25. Valko M, Leibfritz D, Moncola J, CroninMT,Mazura M, Telser J. Review Free radicals and antioxidants in normal physiological functions and human disease. Int J Biochem Cell Biol. 2007;39(1):44–84.

26. Bashan N, Kovsan J, Kachko I, Ovadia H, Rudich A. Positive and negative regulation of insulin signaling by reactive oxygen and nitrogen species. Physiol Rev. 2009 Jan;89(1):27-71.

27. Santiago-López D, Bautista-Martínez JA, Reyes-Hernandez CI, Aguilar-Martínez M, Rivas-Arancibia S. Oxidative stress, progressive damage in the substantia nigra and plasma dopamine oxidation, in rats chronically exposed to ozone. Toxicol Lett. 2010;197(3):193-200.

28. Butterfield DA. Oxidative stress in neurodegenerative disorders. Antioxid Redox Signal. 2006;8(11-12):1971-3.

29. Valko M, Izakovic M, Mazur M, Rhodes CJ, Telser J. Role of oxygen radicals in DNA damage and cancer incidence. Mol Cell Biochem. 2004;266(1-2):37-56

30. Schieber M, Chandel NS. ROS function in redox signaling and oxidative stress. Curr Biol. 2014;24(10):R453-62.

31. Stadtman ER, Levine RL. Protein oxidation. Ann N Y Acad Sci. 2000;899:191–208.

32. Betteridge DJ. What is oxidative stress? Metabolism. 2000;49(2 Suppl 1):3-8.

33. Cooke MS, Evans MD, Dizdaroglu M, Lunec J. Oxidative DNA damage: mechanisms, mutation, and disease. FASEB J. 2003;17(10):1195-214.

34. van der Oost R, Beyer J, Vermeulen NP. Fish bioaccumulation and biomarkers in environmental risk assessment: a review. Environ Toxicol Pharmacol. 2003;13:57–149.

35. Lushchak VI. Environmentally induced oxidative stress in aquatic animals. Aquat Toxicol. 2011;101:13–30.

36. Kanno S, Hirano S, Mukai T, Ro A, Kato H, Fukuta M, Aoki Y. Cellular uptake of paraquat determines subsequent toxicity including mitochondrial damage in lung epithelial cells. Leg Med (Tokyo). 2019;37:7-14.

37. Jaroonwitchawan T, Chaicharoenaudomrung N, Namkaew J, Noisa P. Curcumin attenuates paraquat-induced cell death in human neuroblastoma cells through modulating oxidative stress and autophagy. Neurosci Lett. 2017;636:40-47.

38. Dou T, Yan M, Wang X, Lu W, Zhao L, Lou D, Wu C, Chang X, Zhou Z. Nrf2/ARE Pathway Involved in Oxidative Stress Induced by Paraquat in Human Neural Progenitor Cells. Oxid Med Cell Longev. 2016;2016:8923860.

39. Wang S, Zhang Q, Zheng S, Chen M, Zhao F, Xu S. Atrazine exposure triggers common carp neutrophil apoptosis via the CYP450s/ROS pathway. Fish Shellfish Immunol. 2019;84:551-557.

40. Ansari SM, Saquib Q, Attia SM, Abdel-Salam EM, Alwathnani HA, Faisal M, Alatar AA, Al-Khedhairy AA, Musarrat J. Pendimethalin induces oxidative stress, DNA damage, and mitochondrial dysfunction to trigger apoptosis in human lymphocytes and rat bone-marrow cells. Histochem Cell Biol. 2018;149(2):127-141.

41. Degl'Innocenti D, Ramazzotti M, Sarchielli E, Monti D, Chevanne M, Vannelli GB, Barletta E. Oxadiazon affects the expression and activity of aldehyde dehydrogenase and acylphosphatase in human striatal precursor cells: A possible role in neurotoxicity. Toxicology. 2019;411:110-121.

42. Laville N, Balaguer P, Brion F, Hinfray N, Casellas C, Porcher JM, Aït-Aïssa S. Modulation of aromatase activity and mRNA by various selected pesticides in the human choriocarcinoma JEG-3 cell line. Toxicology 2006;228, 98-108.

43. Kuwata K, Inoue K, Ichimura R, Takahashi M, Kodama Y, Yoshida M. Constitutive active/androstane receptor, peroxisome proliferator-activated receptor α, and cytotoxicity are involved in oxadiazon-induced liver tumor development in mice. Food Chem Toxicol. 2016;88, 75-86.

44. Rahal A, Kumar A, Singh V, Yadav B, Tiwari R, Chakraborty S, Dhama K. Oxidative stress, prooxidants, and antioxidants: the interplay. Biomed Res Int. 2014;2014:761264.

45. Kumar S, Pandey AK. Chemistry and biological activities of flavonoids: an overview. Scientific World Journal. 2013; 2013():162750.

46. Beecher GR. Overview of dietary flavonoids: nomenclature, occurrence and intake. J Nutr. 2003;133(10):3248S-3254S.

47. Spencer JP, Abd El Mohsen MM, Minihane AM, Mathers JC. Biomarkers of the intake of dietary polyphenols: strengths, limitations and application in nutrition research. Br J Nutr. 2008;99(1):12-22.

48. Pandey KB, Rizvi SI. Plant polyphenols as dietary antioxidants in human health and disease. Oxid Med Cell Longev. 2009;2(5):270-8.

49. D'Archivio M, Filesi C, Di Benedetto R, Gargiulo R, Giovannini C, Masella R. Polyphenols, dietary sources and bioavailability. Ann Ist Super Sanita. 2007;43(4):348-61.

50. Nowicka G, Panczenko-Kresowska B. Flavonoids: antioxidative compounds and their role in prevention of ischemic heart disease. Pol Merk Lek. 2003;15(89): 441-444.

51. Nijveldt RJ, van Nood E, van Hoorn DE, Boelens PG, van Norren K, van Leeuwen PA. Flavonoids: a review of probable mechanisms of action and potential applications. Am J Clin Nutr. 2001;74(4):418-25.

52. Braicu C, Pilecki V, Balacescu O, Irimie A, Neagoe IB. The relationships between biological activities and structure of flavan-3-ols. Int J Mol Sci. 2011;12(12):9342-53.

53. Cho HS, Kim S, Lee SY, Park JA, Kim SJ, Chun HS. Protective effect of the green tea component, L-theanine on environmental toxins-induced neuronal cell death. Neurotoxicology. 2008;29(4):656-62.

54. Hou RR, Chen JZ, Chen H, Kang XG, Li MG, Wang BR. Neuroprotective effects of (-)-epigallocatechin-3-gallate (EGCG) on paraquat-induced apoptosis in PC12 cells. Cell Biol Inter. 2008;32(1): 22-30.

55. Kamalden TA, Ji D, Osborne NN. Rotenone-induced death of RGC-5 cells is caspase independent, involves the JNK and p38 pathways and is attenuated by specific green tea flavonoids. Neurochem Res. 2012;37(5):1091-101.

56. Heikal TM, Tawab H, Mossa MA, Abdel Rasoul and Gehan I Kh.,The ameliorating effects of green tea extract against cyromazine and chlorpyrifos induced liver toxicity in male rats. Asian J Pharm Clin Res. 2013;6: 48.

57. Kim HR, Park BK, Oh YM, Lee YS, Lee DS, Kim HK, et al. Green tea extract inhibits paraquat-induced pulmonary fibrosis by suppression of oxidative stress and endothelin-l expression. Lung 2006;184: 287–295.

58. Jung KH, Lee JH, Thien Quach CH, et al. Resveratrol suppresses cancer cell glucose uptake by targeting reactive oxygen species-mediated hypoxia-inducible factor-1α activation. J Nucl Med. 2013;54(12): 2161-2167.

59. Das L, Vinayak M. Long term effect of curcumin in restoration of tumour suppressor p53 and phase-II antioxidant enzymes via activation of Nrf2 signalling and modulation of inflammation in prevention of cancer. PLoS One. 2015;10(4): e0124000.

60. Zhao Y, Fan C, Zhang A, Zhang Y, Wang F, Weng Q, Xu M. Walnut Polyphenol Extract Protects against Malathion- and Chlorpyrifos-Induced Immunotoxicity by Modulating TLRx-NOX-ROS. Nutrients. 2020;12(3):616.

61. Liu H, Wan Y, Wang Y, Zhao Y, Zhang Y, Zhang A, Weng Q, Xu M. Walnut Polyphenol Extract Protects against Fenitrothion-Induced Immunotoxicity in Murine Splenic Lymphocytes. Nutrients. 2018; 10(12):1838.

62. Yang L, Ma S, Han Y, Wang Y, Guo Y, Weng Q, Xu M. Walnut Polyphenol Extract Attenuates Immunotoxicity Induced by 4-Pentylphenol and 3-methyl-4-nitrophenol in Murine Splenic Lymphocyte. Nutrients. 2016; 8(5):287.

63. Haberer G, Kieber JJ. Cytokinins. New insights into a classic phytohormone. Plant Physiol. 2002;128(2):354-62.

64. Barciszewski J, Massino F, Clark BF. Kinetin--a multiactive molecule. Int J Biol Macromol. 2007;40(3):182-92.

65. Olsen A, Siboska GE, Clark BF, Rattan SI. N(6)-Furfuryladenine, kinetin, protects against Fenton reaction-mediated oxidative damage to DNA. Biochem Biophys Res Commun. 1999;265(2):499-502.

66. Dolezal K, Popa I, Hauserová E, Spíchal L, Chakrabarty K, Novák O, Krystof V, Voller J, Holub J, Strnad M. Preparation, biological activity and endogenous occurrence of N6-benzyladenosines. Bioorg Med Chem. 2007;15(11):3737-47.

67. Dolezal K, Popa I, Krystof V, Spíchal L, Fojtíková M, Holub J, Lenobel R, Schmülling T, Strnad M. Preparation and biological activity of 6-benzylaminopurine derivatives in plants and human cancer cells. Bioorg Med Chem. 2006;14(3):875-84.

68. Kim S, Lee J, Jung E, Lee J, Huh S, Hwang H, Kim Y, Park D. 6-Benzylaminopurine stimulates melanogenesis via cAMP-independent activation of protein kinase A. Arch Dermatol Res. 2009;301(3):253-8.

69. Boichot E, Wallace JL, Germain N, Corbel M, Lugnier C, Lagente V, Bourguignon JJ. Anti-inflammatory activities of a new series of selective phosphodiesterase 4 inhibitors derived from 9-benzyladenine. J Pharmacol Exp Ther. 2000;292(2):647-53.

70. Sen P, Chakraborty PK, Raha S. p38 mitogen-activated protein kinase (p38MAPK) upregulates catalase levels in response to low dose H2O2 treatment through enhancement of mRNA stability. FEBS Lett. 2005;579(20):4402-6.

71. English JJr, Bonner J, Haagen-Smit AJ. Structure and synthesis of a plant wound hormone. Science 1939;90: 329.

72. English JJr, Bonner J. The wound hormones of plants. Traumatin, the active principle of the bean test. J Biol Chem.1937;121: 791-799.

73. Jabłońska-Trypuć A, Pankiewicz W, Czerpak R. Traumatic Acid Reduces Oxidative Stress and Enhances Collagen Biosynthesis in Cultured Human Skin Fibroblasts. Lipids. 2016;51(9):1021-35.

74. Dichi I, Breganó JW, Cecchini R. Role of oxidative stress in chronic diseases. CRC Press 2014: 609-642.

75. Jabłońska-Trypuć A, Krętowski R, Wołejko E, Wydro U, Butarewicz A. Traumatic acid toxicity mechanisms in human breast cancer MCF-7 cells. Regul Toxicol Pharmacol. 2019;106:137-146.

76. Jabłońska-Trypuć A, Wydro U, Wołejko E, Butarewicz A. Toxicological Effects of Traumatic Acid and Selected Herbicides on Human Breast Cancer Cells: In Vitro Cytotoxicity Assessment of Analyzed Compounds. Molecules. 2019; 24(9):1710.

77. Jabłońska-Trypuć A, Wydro U, Wołejko E, Rodziewicz J, Butarewicz A. Possible Protective Effects of TA on the Cancerous Effect of Mesotrione. Nutrients. 2020;12(5):1343.
